# Failure of Direct Oral Anticoagulation in Preventing Left Ventricular Thrombus Progression After Myocardial Infarction: A Case Report

**DOI:** 10.3390/reports9010048

**Published:** 2026-02-02

**Authors:** Andreas Merz, Daniel Armando Morris, Henryk Dreger, Ingo Hilgendorf, Matthias Schneider-Reigbert

**Affiliations:** 1 Department of Cardiology, Angiology and Intensive Care Medicine, Deutsches Herzzentrum der Charité, Augustenburger Platz 1, 13353 Berlin, Germany; 2 DZHK (German Center for Cardiovascular Research), Partner Site Berlin, 10785 Berlin, Germany; 3 Charité—Universitätsmedizin Berlin, Corporate Member of Freie Universität Berlin and Humboldt-Universität zu Berlin, 10117 Berlin, Germany

**Keywords:** left ventricular thrombus, direct oral anticoagulation, Vit K antagonist, DOAC

## Abstract

**Background and Clinical Significance**: Left ventricular thrombus formation after acute coronary syndrome represents a severe complication. Comprehensive echocardiographic assessment of the entire ventricle is essential, as regional wall motion abnormalities predispose to thrombus development. Although vitamin K antagonists have traditionally been the cornerstone of therapy, the convenience of direct oral anticoagulants has made them increasingly popular. However, the paucity of prospective data raises concerns regarding their general interchangeability. **Case Presentation:** We present a case of a basal left ventricular thrombus that rapidly progressed in size despite triple antithrombotic therapy including Apixaban. **Conclusions:** Following ACS, regional LV dysfunction predisposes to LVT formation—even in patients with only mild to moderate systolic impairment or non-apical akinesia. Although rare, LVT may also develop in basal and mid-ventricular segments. Anticoagulant selection should remain individualized, and short-term follow-up imaging is necessary to monitor therapeutic response.

## 1. Introduction and Clinical Significance

Acute coronary syndrome (ACS) can lead to various complications, including left ventricular thrombus (LVT) formation. Transthoracic echocardiography (TTE) remains the first-line imaging modality for LVT detection [1]. While apical thrombi following left anterior descending artery (LAD) infarction are by far the most common manifestation, all left ventricular (LV) regions should be carefully assessed for atypical locations, as regional wall motion abnormalities predispose to thrombus formation—even in patients with only mildly to moderately reduced left ventricular ejection fraction (LV-EF) [2]. As a potentially life-threatening condition, anticoagulation must be initiated once LVT is diagnosed. However, prospective data guiding the choice of oral anticoagulant (OAC) for LVT management remain scarce [3,4,5]. Therefore, the OAC regimen should be individualized according to the patient’s clinical profile and serial imaging findings. We present a case of myocardial infarction (MI) complicated by a basal LVT that increased in size despite triple-therapy including Apixaban.

## 2. Case Presentation

A 63-year-old man presented to our hospital with palpitations, shortness of breath (New York Heart Association functional class III–IV), and a sensation of throat tightness that had begun three days earlier. Two weeks before admission, he had experienced severe heartburn (visual analog scale 9/10) accompanied by hyperhidrosis, nausea, and vomiting lasting for two days. His medical history included arterial hypertension and hyperlipoproteinemia. He was an active smoker with a 40 pack-year history and was not taking any regular medication.

The initial electrocardiogram (ECG) revealed sustained ventricular tachycardia. Due to hemodynamic instability, the patient was transferred to the intensive care unit, where electrical cardioversion under sedation successfully restored sinus rhythm. Post-cardioversion ECG demonstrated discrete ST-segment elevation in leads II, III, and aVF, along with discordant T-wave inversion in leads I and aVL. Cardiac biomarkers were moderately elevated.

TTE showed mildly reduced LV-EF of 40–45% with hypokinesia of the basal and mid segments of the anterolateral, inferoseptal, and inferior walls, as well as the mid inferolateral segment. Coronary angiography revealed three-vessel coronary artery disease (CAD). The culprit lesion in the posterolateral branch of the circumflex artery (CX) was successfully dilated, and a drug-eluting stent was implanted. Dual antiplatelet therapy (DAPT) with acetylsalicylic acid (ASA) 100 mg once daily and Ticagrelor 90 mg twice daily was initiated.

**Figure 1:**
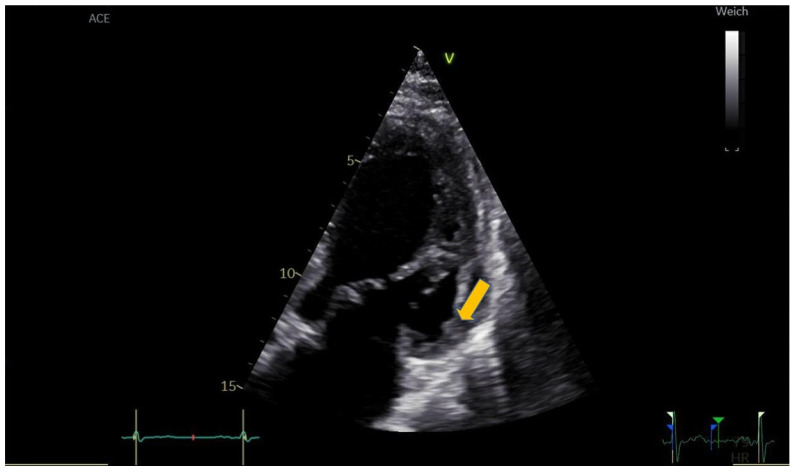
Day 3. Transthoracic echocardiography, modified apical four-chamber view focused on the basal anterolateral wall. A 30 × 8 mm echodense, oscillating structure (arrow) is visible at the basal segment of the anterolateral wall.

On day three after admission, TTE revealed a 30 × 8 mm mobile, echodense, oscillating structure attached to the basal segment of the anterolateral wall, highly suggestive of early thrombus formation (Figure 1, Supplemental Video S1). Direct oral anticoagulant (DOAC) therapy with Apixaban 5 mg twice daily was initiated in addition to DAPT, and Ticagrelor was replaced with Clopidogrel.

**Figure 2:**
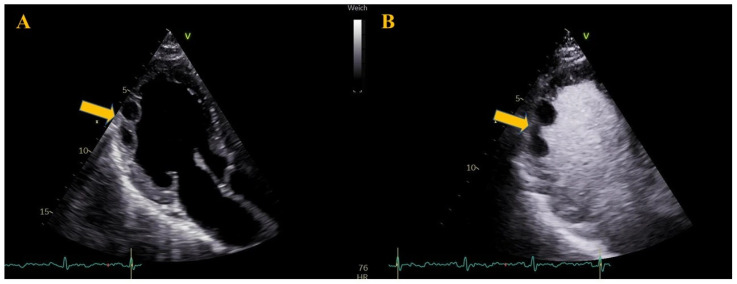
Day 6. Transthoracic echocardiography, apical three-chamber view. Panel (**A**): Thrombus progression despite triple-therapy, now appearing as two mobile masses (14 × 12 mm and 18 × 11 mm) attached to the mid-to-apical anterolateral wall. Panel (**B**): Contrast-enhanced left heart echocardiography demonstrating improved thrombus delineation.

Three days later, contrast-enhanced left heart echocardiography was performed. Despite DOAC in addition to DAPT, the thrombus had increased in size and now appeared as mobile masses located within the akinetic regions of the mid anterolateral, inferolateral, and apical lateral walls (Figure 2, Supplemental Video S2). Anticoagulation was switched to a vitamin K antagonist (VKA. Phenprocoumon, with a loading dose of 9 mg on day 6, 6 mg on day 7, and 3 mg on day 8) with low-molecular-weight heparin (LMWH) bridging at a dose of 1000 IU per 10 kg body weight; ASA was discontinued.

**Figure 3:**
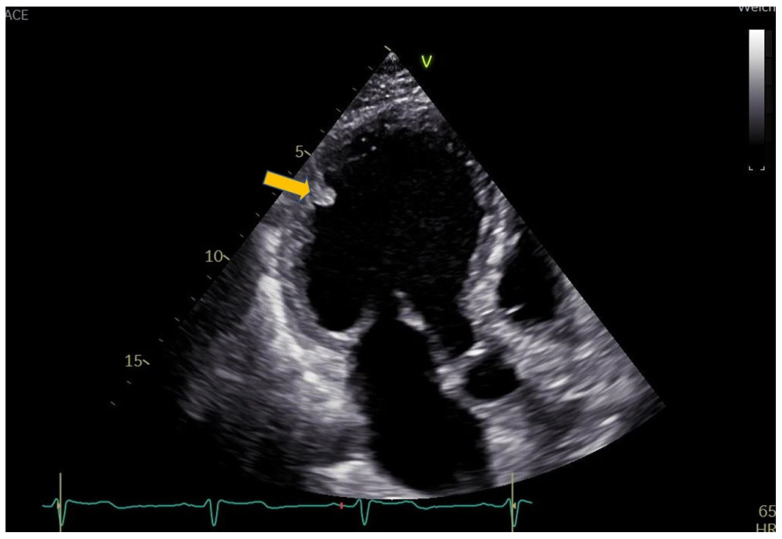
Day 9. Transthoracic echocardiography, apical three-chamber view. Marked reduction in the size of the left ventricular thrombus.

**Figure 4:**
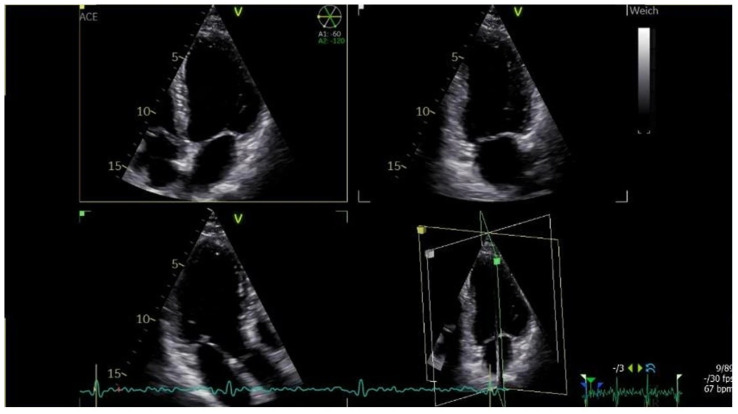
Day 64. Transthoracic echocardiography, triplane view demonstrating the apical four, two-, and three-chamber view. Demonstrating complete resolution of the left ventricular thrombus.

The patient was discharged, and a short-term follow-up was scheduled two days later. TTE at that visit demonstrated a reduction in the LVT (Figure 3). The INR was 6, LMWH was stopped and the VKA dose was adjusted. A subsequent follow-up nine weeks later showed complete thrombus resolution (Figure 4).

## 3. Discussion

Complications of ACS include LVT formation, which is significantly associated with morbidity and mortality [6]. The incidence of LVT following MI has declined in the era of broadly accessible primary percutaneous coronary intervention [7]. Nevertheless, it remains a relatively common complication, occurring in up to 15% of patients with ST-elevation myocardial infarction (STEMI) [8], particularly among those who present days or even weeks after symptom onset, as in the case presented here. Thrombus formation can be explained by Virchow’s triad: in ACS, the combination of blood stasis due to regional wall motion abnormalities and subendocardial tissue injury from ischemia both contribute to LVT development [7,9]. Among published predictors, LV dysfunction is the strongest independent factor for post-MI LVT formation, with significantly lower LV-EF observed in patients who develop LVT [6,10]. Additional risk factors include infarct size, apical asynergy, LV aneurysm, and anterior-apical scar [11,12,13,14]. While most LVTs occur apically, 11% have been reported at the septal wall and 3% at the inferolateral wall [15]. The presence of LVT closely correlates with the LV region exhibiting the greatest functional impairment [13].

Cardiac magnetic resonance imaging (CMR) is considered the gold-standard modality for LVT assessment [2]. However, echocardiography remains the most widely used first-line diagnostic tool because of its broad availability and cost-effectiveness [6,16]. Unlike left atrial masses or thrombi in the left atrial appendage, transesophageal echocardiography provides little additional value over TTE for LVT diagnosis, as the LV apex is typically difficult to visualize [17]. TTE demonstrates a specificity of 95–98% for detecting LVT after acute MI [17,18], though its sensitivity (21–35%) can be limited by suboptimal acoustic windows, inadequate visualization of the LV apex, or small thrombus size [2,9,17]. Both specificity and sensitivity can be improved by contrast echocardiography, reaching 99% and 64%, respectively [2]. Common causes of false-positive findings include artifacts, prominent trabeculations, or a tangentially imaged LV wall [19,20].

In the presented case, corresponding to the culprit lesion in the CX artery, the entire anterolateral wall was akinetic three days after admission, and an echodense structure was visible at its basal segment. LVT prevalence in non-anterior MI has been shown to increase with extension of inferior necrosis toward the posterolateral wall [21], as observed in our patient, who demonstrated medial and apical inferolateral akinesia.

Once diagnosed, therapy should be initiated promptly, as LVT formation is associated with a 22% risk of embolic events [22]. Although treatment has traditionally centered on VKAs, DOACs have become increasingly attractive. However, existing guidelines lack randomized controlled trials (RCTs) comparing optimal OAC regimens and concomitant antiplatelet therapy in post-MI LVT. A recent meta-analysis including 11 studies with approximately 15,000 patients reported that DOACs were associated with higher rates of LVT resolution (*p* = 0.04), lower rates of stroke and systemic embolism (*p* < 0.01), and reduced major and bleeding events (*p* = 0.05) compared with VKA in patients with post-MI LVT [23]. Similar results have been published [24,25]. DOACs were found to be non-inferior to VKAs in three smaller RCTs [3,4,5]. The American Heart Association considers DOACs a reasonable alternative to VKAs [7], whereas the European Society of Cardiology recommends that either drug class may be considered for LVT treatment [1]. Given the easier handling and fewer monitoring requirements of DOACs relative to VKAs, our patient was initially treated with a DOAC.

The timing of imaging relative to ACS appears crucial, as most LVTs do not form immediately after the event. While LVTs occur earlier in patients with an initial LV-EF ≤ 40% or multivessel CAD, the highest detection rates have been reported approximately two weeks after the index event [26,27,28,29,30]. This suggests that LVTs may be missed in case of early hospital discharge and underscores the importance of follow-up imaging in high-risk patients without initial thrombus. For assessing thrombus resolution, repeat imaging at three months is recommended [6,7].

In the present case, it is likely that the patient experienced a STEMI two weeks before admission. TTE after three days of triple antithrombotic therapy revealed further LVT enlargement. Persistence or progression of LVT despite adequate OAC, as observed here, remains poorly studied. In accordance with consensus-based recommendations [7], our patient’s anticoagulation therapy was switched from DOAC to VKA. In a cohort of 159 patients undergoing serial echocardiography, complete thrombus resolution was observed in 62.3% after a median of 103 days, while recurrence or progression occurred in 14.5% [31]. A multicenter cohort study on anticoagulation strategies for LVTs included a substantial number of patients who switched therapy from DOAC to VKA or vice versa [32]. Common reasons for switching from VKA to DOAC included patient convenience, whereas cost considerations mainly prompted changes from DOAC to VKA. Sensitivity analyses for embolic events revealed no significant differences between groups. Further studies are warranted to clarify the mechanisms and optimal management of LVT persistence or progression.

The presented case highlights the importance of recognizing atypically located LVT, performing serial TTE follow-up, and recognizing the possibility of early progression despite OAC, thereby necessitating individualized anticoagulant selection.

## 4. Conclusions

Following ACS, regional LV dysfunction predisposes to LVT formation—even in patients with only mild to moderate systolic impairment or non-apical akinesia. Although rare, LVT may also develop in basal and mid-ventricular segments. Comprehensive echocardiographic assessment of all LV regions is therefore essential. Early and follow-up imaging after ACS are crucial for timely LVT detection, particularly in patients presenting late after symptom onset. The assumption of general interchangeability between VKAs and DOACs cannot be supported. Anticoagulant selection should remain individualized, and short-term follow-up imaging is necessary to monitor therapeutic response.

## Data Availability

The original data presented in the study are included in the article, further inquiries can be directed to the corresponding author.

## Supplementary Materials

The following supporting information can be downloaded at: https://www.mdpi.com/article/10.3390/reports9010048/s1, Supplementary Video S1: On day three after admission, TTE revealed a 30 × 8 mm mobile, echodense, oscillating structure attached to the basal segment of the anterolateral wall, highly suggestive of early thrombus Formation; Supplementary Video S2: Three days after the initial echo, contrast-enhanced left heart echocardiography was performed. Despite DOAC in addition to DAPT, the thrombus had increased in size and now appeared as mobile masses located within the akinetic regions of the mid anterolateral, inferolateral, and apical lateral walls.
